# The Potential of Traditional Norwegian KVEIK Yeast for Brewing Novel Beer on the Example of Foreign Extra Stout

**DOI:** 10.3390/biom11121778

**Published:** 2021-11-26

**Authors:** Joanna Kawa-Rygielska, Kinga Adamenko, Witold Pietrzak, Justyna Paszkot, Adam Głowacki, Alan Gasiński, Przemysław Leszczyński

**Affiliations:** 1 Department of Fermentation and Cereals Technology, Wrocław University of Environmental and Life Sciences, Chełmońskiego 37, 51-630 Wrocław, Poland; kinga.adamenko@upwr.edu.pl (K.A.); witold.pietrzak@upwr.edu.pl (W.P.); justyna.paszkot@upwr.edu.pl (J.P.); adam.glowacki@upwr.edu.pl (A.G.); alan.gasinski@upwr.edu.pl (A.G.); 2 Department of Biology and Medical Parasitology, Wrocław Medical University, Mikulicza-Radeckiego 9, 50-367 Wrocław, Poland; przemyslaw.leszczynski@umw.edu.pl

**Keywords:** special microorganisms, Norwegian KVEIK-type yeast, brewing technology, ethanolic fermentation, HPLC, GC/MS

## Abstract

The development of craft brewing has spurred huge interest in unusual and traditional technologies and ingredients allowing the production of beers that would fulfil consumers’ growing demands. In this study, we evaluated the brewing performance of traditional Norwegian KVEIK yeast during the production of Foreign Extra Stout beer. The content of alcohol of the KVEIK-fermented beer was 5.11–5.58% *v*/*v*, the extract content was 5.05–6.66% *w*/*w*, and the pH value was 4.53–4.83. The KVEIK yeast was able to completely consume maltose and maltotriose. The mean concentration of glycerol in KVEIK-fermented beers was higher than in the control sample (1.51 g/L vs. 1.12 g/L, respectively). The use of KVEIK-type yeast can offer a viable method for increasing the concentration of phenolic compounds in beer and for boosting its antioxidative potential. The beers produced with KVEIK-type yeast had a total phenol content of 446.9–598.7 mg GAE/L, exhibited antioxidative potential of 0.63–1.08 mM TE/L in the DPPH^•^ assay and 3.85–5.16 mM TE/L in the ABTS^•+^ assay, and showed a ferric ion reducing capacity (FRAP) of 3.54–4.14 mM TE/L. The KVEIK-fermented bears contained various levels of volatile compounds (lower or higher depending on the yeast strain) and especially of higher alcohols, such as 3-metylobutanol, 2-metylobutanol, and 1-propanol, or ethyl esters, such as ethyl acetate or decanoate, compared to the control beers. In addition, they featured a richer fruity aroma (apricot, dried fruit, apples) than the control beers fermented with a commercial US-05 strain.

## 1. Introduction

The Norwegian yeast strains of the KVEIK type can be divided into two groups according to their geographic origin, i.e., those from the north or south of the Jostedal glacier. The first group derives from the regions of Voss, Stranda, Granvin, and Laedal, while the second group from the regions of Hornindal, Stordal, and Sykkylven. In Scandinavian countries, these yeast strains have been for years used locally to produce traditional beers, such as konnjøl, stjørdalsøl, or maltøl [1]. The KVEIK yeast is still understudied. It has been shown to differ phenotypically from conventional top-fermenting brewing yeast, and its distinguishing features include the following: resistance to high temperatures and fermentation ability at temperatures above 30 °C, high rate of alcohol fermentation, strong flocculation, high tolerance to ethyl alcohol, low production of 4-vinylguaiacol, no phenolic aftertaste, and the development of a strong and aromatic odor profile [2,3,4,5]. Its traditional storage method is also interesting. The yeast suspension is first dipped in an oval-shaped wooden construction, then in ash or flour, and finally dried. The first constructions of this type date back to the onset of the 17th century [6].

There are only a few scientific papers in which the authors studied KVEIK yeast. These include works where the KVEIK yeast was analyzed in terms of its genetic pool [1,7] or its potential for the commercial production of Norwegian historical beers [8]. There is only one study that determined the possibility of using KVEIK yeast in fermentation processes, but not in brewing. Its authors used a selected yeast strain to produce fermented beverages based on acid whey [9]. Considering the above findings, the aim of the research described in this manuscript was to establish the potential of using traditional Norwegian KVEIK-type yeast for the production of novel beers through the example of Foreign Extra Stout. Beer of this style is dark, dry, and strong. It has the taste and aroma of roasted beans, coffee, and chocolate, a low to moderate hop aroma, and a medium degree of carbonation. Our research responds not only to the paucity of literature data on this subject, but also to the needs of the fermented beverage market. The beer revolution around the world has led to the establishment of a large number of small- and medium-sized craft breweries, which seek innovative technologies to obtain new features in their finished products. One of the methods is to use Norwegian KVEIK yeast, which is potent to produce a broad spectrum of aromatic compounds.

## 2. Materials and Methods

### 2.1. Materials

Oatmeal (Twój Browar, Wrocław, Poland)—added to beers such as white beers, grand cru, or lambic. It contributes to the full flavor, expressive taste, and a creamy consistency of the beer. It is mashed together with malts in the basic hopper. It is recommended to boil the flakes in a separate pot for approximately 30 min and, after cooling, add them to the mash.

Wheat flakes (Twój Browar, Wrocław, Poland)—as an alternative to wheat malt, they provide a pleasant sweetness of wheat in the finished beer, increase the body, and improve foam. They are mashed together with malts in the basic hopper. It is recommended to boil the flakes in a separate pot for approximately 30 min and, after cooling, add them to the mash.

Roasted barley (Viking Malt, Strzegom, Poland)—roasted good-quality malting barley grains. The roasting process is similar to the dyeing malt process, with special care taken not to burn the grain. The final temperature is over 200 °C. The taste is sharper and more bitter than that of the colored malt. It is used in dark beers, such as stouts and porters, mainly because of its strong color properties. Roasted barley imparts a dry flavor and a distinct color. It is made of 2-row malted barley. Its color is higher than 900 EBC (European Brewery Convention) units. It is an enzymatically inactive malt, and its maximum share in the charge may be up to 10%.

CaraAroma malt (Weyermann, Bamberg, Germany)—special dark caramel malt. It is used for dark beers. Its color range is 300–400 EBC. Its recommended share in the charge should not exceed 15%. It is mainly characterized by caramel and bread aromas, and is made of high-quality barley. It improves mouthfeel, imparts a deep red color, and strengthens flavor stability.

Munich malt (Viking Malt, Poland)—used to emphasize the full and malty flavor, has a color range of 14–18 ECB. It imparts an amber aroma and color to beer. Its nutty and aromatic character is developed within drying at temperatures of 110–120 °C. It is characterized by low enzyme activity compared to Pilsner malt.

Pale Ale malt (Viking Malt, Poland)—color range is 4–7 EBC. It imparts a deep malty flavor and a golden color to the wort. It is commonly used to brew many kinds of pale ale and also for color correction in brewing classic lagers.

Lubelski hops—classified as aromatic hops. The alpha acid content is 3%. It is characterized by tea, lemon, curry, dill, and juniper aromas, as well as featuring floral and herbal notes. It has been cultivated in Poland since 1964 and comes from the Czech Saaz variety. The T90-type pellets of Lubelski hops were used in this study.

Marynka hops—the bitter hops. The content of alpha acid is 6.5%. It is characterized by citrus aromas and floral, spicy, and herbal notes. Moreover, it has a high content of bitter compounds. This hop variety was also used in T90-type pellets during wort boiling.

*Saccharomyces cerevisiae* SafAle US-05 (US-05)—American top-fermenting yeast (Fermentis, Marq en Baroeul, France). It is characterized by a medium level of flocculation and by producing a small amount of diacetyl under its optimal fermentation temperature, which ranges from 18 to 28 °C. It is used to produce a dry and balanced beer with a pure profile.

Horniwdak Var Kveik (HVK)—yeast isolated by Terje Raftevold from Hornindal in Norway (Omega Yeast, Chicago, IL, USA). It is characterized by producing a high amount of tropical fruity aroma and flavor: fresh pineapple, mango, and mandarin. It is used for wort fermentation at the temperatures of 22–37 °C.

*Saccharomyces cerevisiae* FM 53 Voss Kveik (FM53)—yeast strain created and isolated by Sigmund Gjernes from Voss in Norway (Omega Yeast, Chicago, IL, USA); purchased commercially.It is a top-fermenting yeast able to produce fruity aromas: orange, banana, pineapple, peach, and spicy (similar to the Belgian ones). It is characterized by a low attenuation limit (68–80%), giving a sweet and full beer. The temperature of fermentation ranges from 20 to 40 °C.

Var Kveik 2 (VK2)—yeast strain brought from Omega Yeast, Chicago, IL, USA, stored on the agar slant.

Lida Kveik (LK)—yeast from Grodås in Norway (Omega Yeast, Chicago, IL, USA). It is characterized by a fruity and delicate profile with hints of caramel and milk. It is stored on the agar slant.

### 2.2. Methods

#### 2.2.1. Yeast Propagation

A pale ale malt wort with the extract content of 7° Plato was used for inoculum propagation, which was conducted under laboratory conditions. After mashing, the wort was first cooled and then poured into tubes and flasks under sterile conditions. The yeast strains stored on the agar slant were transferred into tubes containing 5 mL of wort. Dry yeast was rehydrated in sterile physiological fluid and then transferred (0.5 mL) into the tubes with the wort. The tubes were shaken for 24 h (250 rpm). The propagated yeasts were transferred into 100 mL Erlenmeyer flasks with 50 mL of the wort. Then, the flasks were shaken again for 24 h (250 rpm). Afterwards, the yeast suspension was transferred from 100 mL flasks into 1000 mL flasks containing 500 mL of the wort. The yeasts were incubated for 24 h (800 rpm).

#### 2.2.2. Wort Preparation

The mashing process was carried out in a 40 L mash tub. High diastatic malts were used; therefore, single-temperature mashing was carried out in which the two main enzymes, alpha and beta amylase, were active. Oat (0.2 kg) and wheat flakes (0.5 kg) were poured with 10 L of water, and then heated to 100 °C and boiled for 20 min to gelatinize the starch. Afterwards, roasted barley (0.4 kg), CaraAroma malt (0.8 kg), Munich malt (2.0 kg), Pale Ale malt (6.0 kg), and 17 L of water were added, and the mashing process was started at 67 °C and continued for 90 min. Afterwards, an iodine test was performed to check if the starch was hydrolyzed. Then, the mash temperature was raised to 78 °C and maintained for 10 min to denature the enzymes. Then, the saccharified mash was transferred to a filter tank consisting of a drain cock tap and steel braid; 14 L of water with a temperature of 78 °C was used for filtration. The 30 L of wort obtained was boiled with hops at the temperature of 100 °C. Then, 85 g of Marynka hops was boiled for 70 min and 30 g of Lubelski hops was added for the last 10 min of boiling. The hopped wort was cooled using a stainless-steel immersion cooler. A sample of the wort was also taken and cooled to 20 °C to control and adjust the extract content to 15° Plato using an optical refractometer. The wort was divided into five variants; each of the 6 L were divided again for three replications. All worts were inoculated with four different strains of KVEIK yeast and a commercial US-05 yeast using 500 mL of the inoculum. The primary fermentation was carried out for 7 days at 25 °C in a cooling incubator. Afterwards, young beer was taken over the yeast sediment. The secondary fermentation lasted 14 days at 25 °C. In the next stage, the young beer was poured into 0.5 L bottles, to which 5 g glucose/L was added. All beers were aged for 21 days at 25 °C.

#### 2.2.3. Basic Physicochemical Parameters and pH Measurements

Ethyl alcohol content, degree of fermentation, energy value, extract content, as well as density were measured with an Anton Paar Alex 500 oscillating density meter coupled with a near infrared spectroscope for measuring ethanol concentration (Anton Paar, Buchs, Austria). Beers were degassed, centrifuged (2675 centrifugal force (RCF), 6000 rpm, 10 min), and filtered with diatomaceous earth (1 g/100 mL) on laboratory filter paper. Their pH value was measured using a Mettler Toledo MP 240 pH meter (Columbus, OH, USA). Analyses were carried out in three replications.

#### 2.2.4. High-Performance Liquid Chromatography (HPLC)

The concentrations of dextrins, glucose, maltose, maltotriose, glycerol, and acetic and lactic acids in the beer samples were determined with high-performance liquid chromatography. Degassed and centrifuged (2675 centrifugal force (RCF), 6000 rpm, 10 min) beers were double-diluted with double-distilled water and filtered through nylon filters (0.22 µm) to chromatographic vials. Beer samples were analyzed using a Prominence liquid chromatograph (Shimadzu, Kyoto, Japan) equipped with a Rezed ROA-Organic Acid H^+^ column (300 × 4.6 mm; Phenomenex, Torrance, CA, USA). Measurement parameters were as follows: elution temperature 60 °C, injection volume 20 μL, flow rate 0.6 mL/min, thermostat refractometric detector 50 °C, and mobile phase 0.005 M H_2_SO_4_. Concentrations of the analyzed compounds were determined based on a five-point calibration curve integrated in Chromax 10.0 software (Pol-Lab, Wilkowice, Poland). Analyses were carried out in three replications.

#### 2.2.5. Total Polyphenol Content (TPC)

The Folin–Ciocalteu spectrophotometric method [10] was used to analyze the total polyphenol content. A sample of beer/wort in the amount of 0.1 mL and 0.2 mL Folin–Ciocalteu reagent were mixed in a plastic cuvette and incubated for 3 min at 25 °C. Afterwards, 1 mL of 20% Na_2_CO_3_ solution and 2 mL of redistilled water were added to the sample, which was incubated in the dark for 1 h at 25 °C. The absorbance was measured at a wavelength of 765 nm. Results were expressed as gallic acid equivalents (GAE) per 100 mL of beer or wort. Analyses were carried out in three replications. Calibration curves of gallic acid in the range of 0.30–9.00 mg GAE/L were used to read the results.

#### 2.2.6. Antioxidative Activity Assayed with the DPPH^•^ Reagent

The antioxidative activity was measured by the method with the DPPH^•^ reagent (Yen and Chen, 1995). A sample of beer or wort (0.1 mL) was mixed in a polystyrene cuvette with 2 mL of 0.04 mmol/L DPPH^•^ ethanolic solution and 0.4 mL of redistilled water. The sample was incubated for 10 min at room temperature. Absorbance was measured at a wavelength of 517 nm. Results were expressed as Trolox equivalents (TE) per 1 L of beer or wort (mmol TE/L). Analyses were carried out in three replications. Calibration curves in the range of 2–10 µmol TE/L showed good linearity (r^2^ ≥ 0.998).

#### 2.2.7. Antiradical Activity Assayed with ABTS^•+^

Another method to analyze the antioxidative activity is that with ABTS cation radical reduction [11]. A sample of beer or wort (0.03 mL) was mixed in a plastic cuvette with 3 mL of an ABTS^•+^ solution (absorbance of the ABTS^•+^ solution measured at a wavelength of 734 nm reached 0.700). Absorbance of the samples was measured after 6 min of incubation at 25 °C. Results were expressed as Trolox equivalents per 1 L of beer or wort (mmTE/L). Analyses were carried out in three replications. Calibration curves in the range of 1.70–21.70 µmol TE/L showed good linearity (r^2^ ≥ 0.999).

#### 2.2.8. Ability of Ferric Ion Reduction (FRAP)

The ability to reduce ferric ions was determined using the FRAP method [12]. The ferric reducing antioxidant power (FRAP) reagent was prepared by combining 20 mL of an aqueous solution of iron (III) chloride (0.1018 g FeCl_3_) with a solution of 2,4,6-Tris(2-pyridyl)-*s*-triazine (0.0664 g TPTZ) in 40 mM hydrochloric acid (20 mL HCl) with 20 mmol/L ferric chloride in acetate buffer (pH 3.6). A sample of beer of wort (1 mL) was mixed in a plastic cuvette with 3 mL of the FRAP reagent. Absorbance was measured at 593 nm. The results are presented as the mean of triplicates in mmol of Trolox/L of beer or wort. Analyses were carried out in three replications. Calibration curves in the range 1.25–12.50 µmol TE/L showed good linearity (r^2^ ≥ 0.998).

#### 2.2.9. Analysis of Volatile Compounds

Volatile compounds of the tested beers were analyzed by the gas chromatography technique coupled with flame ionizing detection (GC–FID), using a GC2010 Plus apparatus with a FID-2010 and a headspace autosampler (HS-20) (Shimadzu Corporation, Kyoto, Japan), equipped with a CP-WAX 57 CB column (50 m × 0.32 mm ID × 0.2 µm) (Agilent Technologies, Santa Clara, CA, USA), by [13]. Beer samples were degassed, mixed with diatomaceous earth (1 g per 100 mL of beer), and filtered through a paper filter. After filtration, 10 mL of beer was transferred to a 20 mL headspace vial. Each vial was conditioned in a headspace autosampler oven set at 40 °C and equilibrated for 20 min at shaking level 2 prior to the injection of the sample into the column. The volume of the volatiles transferred to the column was 1 mL, pressurizing time was 0.5 min, pressurizing equilibration time was 0.1 min, load time was 0.5 min, load equilibrium time was 0.1 min, injection time was 0.5 min, needle flush time was set to 0 min, and total GC cycle time was 60 min. Injection mode was set to split (split ratio 10), and the GC temperature program was as follows: 40 °C, hold 3 min, increase to 80 °C at the rate 5 °C per min, hold 3 min, increase to 140 °C at the rate of 10 °C per min, hold 9 min, increase to 160 °C at the rate of 20 °C, hold 4 min (total program time 34 min). Starting pressure was set at 100 kPa, starting flow was set at 6.6 mL/min, starting column flow was set at 0.33 mL/min, starting linear velocity was set at 11.8 cm/s, and purge flow was set at 3 mL/min. Helium was used as the carrier gas. FID operated at 280 °C at a sampling rate of 40 ms with the stop time at 34 min. H_2_ flow to the FID at the rate of 50 mL/min, air flow rate was 400 mL/min, and makeup gas (helium) flow rate was 30 mL/min. Data were integrated and quantitated in the LabSolutions software (Shimadzu Corporation, Kyoto, Japan). Automatic integration was performed under the following conditions: peak width equal to 3 s, slope at least 1000 uV/min, min. area 1000 counts. Identification of the compounds was performed using analytical standards, with an identification method based on absolute retention time. Quantitation was performed using external standards, with 5 calibration points on the curve (coefficient of determination R^2^ was equal to at least 0.999).

#### 2.2.10. Sensory Analysis

A total of 15 persons participated in the study. The sensory evaluation of all beers was performed in terms of clarity, foaminess, color, bitterness, saturation, taste, and aroma. Scores were given for individual features on a scale of 2–5, where 2 meant the lowest and 5 the highest. Furthermore, the panelists used a separate descriptive form to evaluate aroma (malt, hoppy, ester, other aromas), bitterness (quality, intensity, finish), and taste. The form was devised according to the Polish Association of Home Brewers (PSPD). Assessment was carried out in a sensory analysis laboratory equipped with special individual boxes with standardized color and light.

#### 2.2.11. Statistics

Data were processed using Statistica 13.5 software (StatSoft, Tulsa, OK, USA), based on ANOVA (α = 0.05). Duncan’s test was used to analyze differences between mean results (*p* < 0.05). The tables show values of standard deviation.

## 3. Results and Discussion

### 3.1. Basic Physicochemical Parameters

The physicochemical parameters of the beers, including ethyl alcohol content, real and apparent extract content, density, real and apparent fermentation degree, energy value, and pH value, are presented in Table 1. They were analyzed at three stages of the production process: after primary fermentation, after secondary fermentation, and after beer aging in bottles.

At the primary fermentation stage, the beer fermented using the US-05 yeast strain had the highest ethyl alcohol concentration, which reached 4.51% and was higher by 0.80 percentage points (p.p.) than in the KVEIK-fermented beers. The alcohol content of the KVEIK-fermented beers ranged from 3.56% *v/v* (HVK) to 3.87% *v/v* (VK2). After secondary fermentation, no significant differences were noted in the fermentation degree between the samples, and the mean value of the real fermentation degree determined at this stage reached 49.85%. A study conducted by Preiss et al. [1] has demonstrated that KVEIK yeast features higher fermentation dynamics than commercial yeast strains. However, in their study, the samples were fermented at higher temperatures than in the present research, which may point to a higher temperature optimum of KVEIK yeast compared to the standard yeast strains. The pH value of all beer samples was similar and reached pH = 5.05 on average. The beer sample fermented using the commercial yeast strain had the highest energy value, reaching 48.79 kcal/100 mL. The analysis of the energy value of the KVEIK-fermented beers showed no significant differences between them; their mean energy value was 43.93 kcal/100 mL.

After secondary fermentation, the ethyl alcohol content equalized in all samples and reached 5.21% *v/v* on average. The real fermentation degree was the highest in the US-05 sample (51.94%), whereas in the KVEIK-fermented beers, it ranged from 50.88% in VK2 beers to 51.21% in FM53 beers. The US-05 beer had the highest energy value (46.74 kcal/100 mL), whereas the lowest value of this parameter was determined in the HVK sample (39.08 kcal/100 mL). During secondary fermentation, a tangible decrease was noted in the pH values of the KVEIK-fermented samples. The greatest pH decrease was determined in the HVK and FM53 samples, i.e., by 0.53 and 0.51, respectively, compared to the previous fermentation stage. In turn, the pH value of the US-05 sample decreased by 0.21.

After the stage of beer aging in bottles, the ethyl alcohol concentration increased significantly in the LK sample, i.e., by 0.33 p.p. compared to the value measured after secondary fermentation. In addition, this beer had the highest ethyl concentration of all beer samples tested (5.58% *v*/*v*). Such a significant increase in alcohol content was not noted in the remaining samples. The LK yeast strain featured the highest fermentation degree, reaching 62.51%, whereas the extract content of the beer fermented with this strain was at 5.05% *w*/*w*. The highest fermentation degree was obtained in the study using HVK yeast (52.75%). The mean fermentation degree of the KVEIK-fermented beers was similar to that of the control beer. In addition, the KVEIK-fermented beers had a lower energy value than the control sample. The lowest energy value was determined in the LK beer (41.8 kcal/100 mL), whereas the highest one was obtained in the US-05 beer (49.78 kcal/100 mL). The mean energy value of the KVEIK-fermented beers was 6 kcal/100 mL lower compared to the control US-05 sample.

After aging in bottles, a significant pH value increase was noted in the LK, VK2, and US-05 beers compared to the previous production stage. The lowest final pH value was measured in the HVK sample (pH = 4.53), whereas the highest one was found in the US-05 sample (pH = 4.83). The mean pH value of the KVEIK-fermented beers was 0.16 lower that the pH value of the control sample.

### 3.2. High-Performance Liquid Chromatography (HPLC)

Table 2 presents the results of the HPLC analysis of beers produced with KVEIK and US-05 yeast strains. The beers were analyzed for the content of dextrins (DP4+), maltotriose, maltose, and glucose, being products of the enzymatic hydrolysis of starch. Analyses were conducted at three stages of the production process. The beers were also assessed for their concentrations of glycerol and organic acids (lactic and acetic acids).

The carbohydrate profile analysis demonstrated that all yeast strains tested consumed maltose available in the wort as soon as in the primary fermentation stage. Maltotriose was also completely consumed during the primary fermentation in all beer samples except for the VK2 beer, where its content was 2.74 g/L. The ability to consume maltotriose is a technologically significant feature of brewer’s yeast [1]. Preiss et al. [1] also determined a higher degree of maltotriose fermentation by KVEIK yeast.

The content of dextrins decreased negligibly in all beer samples, with the greatest decrease noted in the HVK sample (39.73 g/L, decrease by 5.63%). At this technological stage, the lowest degree of dextrin fermentation (2.02%) was demonstrated for the VK2 strain (DP4+ content of 41.25 g/L). The mean fermentation degree of dextrins after the primary fermentation reached 3.26%.

After the secondary fermentation stage, the degree of dextrin fermentation differed significantly among the beer samples. The greatest decrease in dextrin content was determined in the FM53 sample (it reached 30.21% compared to the previous stage). At this stage, also the HVK strain showed a high degree of dextrin consumption (29.66% compared to the previous stage). In turn, the LK strain was able to ferment 3.84% of the oligosaccharides (DP4+), whereas their concentration was not decreased by the VK2 strain. At this production stage, the control strain (US-05) fermented 15.04% of dextrins. After the secondary fermentation, the content of these saccharides in the control beer reached 35.02 g/L.

The glycerol concentration varied significantly at the particular stages of the beer production process. The analysis of the samples after the primary fermentation demonstrated the highest glycerol concentration in the VK2 (1.45 g/L) and FM53 (1.43 g/L) samples. The HVK, LK, and US-05 yeast strains produced less glycerol at the beginning of fermentation; its mean concentration reached 1.30 g/L. During secondary fermentation, the glycerol concentration decreased in the following beer samples: HVK (by 24.4% compared to the previous stage), LK (by 5.3%), and US-05 (by 15.3%). In turn, in the FM53 and VK2 samples, its concentration was similar to that from the previous stage. At the stage of beer aging in bottles, the glycerol concentration increased in HVK (by 43.8%), LK (by 28.8%), and VK2 (by 15.3%) samples, and decreased in the FM53 sample (by 4.14%). The concentration of glycerol determined at this production stage did not differ significantly from its concentration determined in the previous production stage. The mean concentration of glycerol in the KVEIK-fermented beers was higher than in the control US-05 sample (1.51 g/L vs. 1.12 g/L, respectively). The concentration of glycerol in beers depends, among other factors, on the yeast strain used and the conditions of the fermentation process. A higher glycerol concentration affects the sensory characteristics of the finished product as it intensifies the perception of a sweet taste and increases product viscosity [14]. Acetic and lactic acids were not detected in any of the beer samples, which indicates the incapability of the yeast strains tested for their production and a lack of bacterial contamination.

### 3.3. Total Polyphenol Content and Antioxidative Activity

The antioxidative properties of beers are due to, among others, phenolic compounds of malt and hops [15]. In order to enhance the bio-activity of beer, attempts are undertaken to enrich it with phenolic compounds by modifying the production technology. The analysis of the impact of biological material on the content of phenolic compounds and antioxidative properties of beers seems to be an interesting avenue of research.

Beer wort had the lowest total polyphenol content (TPC) and exhibited the lowest DPPH^•^ and ABTS^•+^ scavenging ability and the lowest ferric ion reducing capacity (FRAP) among all samples tested (Table 3). The primary fermentation led to a significant increase in the values of all the aforementioned parameters. At this production stage, the KVEIK HVK, VK, and FM53 yeast strains enabled the production of beers having a higher total polyphenol content compared to the control US-05 sample.

The highest DPPH^•^ antioxidative capacity and FRAP value were determined for the VK2 beer. In terms of the ABTS^•+^ scavenging activity determined at this production stage, the KVEIK-fermented beers did not differ significantly from the control sample. After fermentation, all KVEIK beers had a higher TPC than the control US-05 beer. At this stage of the beer production process, the antioxidative activity of the beers varied depending on the yeast strain and analytical method employed. The HVK, LK, and FM53 samples did not differ significantly regarding the DPPH^•^ scavenging activity compared to the control US-05 sample. In turn, the VK2 sample showed lower DPPH^•^ scavenging activity than the control beer. After secondary fermentation, the ABTS^•+^ capacity of beers fermented using the KVEIK HVK, LK, and VK2 strains was higher compared to the control sample. There were no statistically significant differences in the ferric ion reducing capacity between the beers fermented using the biomass of HVK, LK, and FM53 yeast and the control beer fermented with the US-05 strain. After secondary fermentation, a higher FRAP value was determined for the VK2 sample.

Ethanolic fermentation leads to changes in the content of phenolic compounds. These changes are mainly due to their absorption by the yeast biomass and their transformations observed during ethanolic fermentation. Cortese et al. [16] have demonstrated that brewer’s yeast biomass contains more phenolic compounds after fermentation than before it. Moreover, yeast’s capability to absorb phenols depends on the strain used in the fermentation process. The major phenolic compounds identified by these authors in the biomass of fresh brewer’s yeast were quercetin and gallic acids. After fermentation, the biomass had increased content of colupolone, cohumulone, humulone, isoxanthohumol, and trans-ferulic acid, which is indicative of cells’ capability to absorb these compounds from beer wort [16]. Yeast strains are also capable of converting glycosidic forms of phenolic compounds to aglycones [17], which in turn facilitates their easier post-fermentation identification in the medium.

The finished beers had a significantly lower TPC compared to the beers analyzed after primary and secondary fermentation. The content of phenols in the control US-05 beer and HVK beer did not differ significantly from their content in the wort. Despite the TPC decrease at this technological state, it is noteworthy that the beers produced using the KVEIK yeast, i.e., LK, VK2, and FM53, had a statistically higher TPC than the control beer. The highest TPC was determined in the FM53 beer, which was additionally characterized by the highest FRAP value. Similar to after the secondary fermentation, the samples with the highest TPC did not exhibit the highest antioxidative activity determined in the DPPH^·^ and ABTS^•+^ assays. In these assays, the distinguishing radical scavenging activity was found for the HVK beer, which had the lowest TPC among all beers after aging.

Turbidity (haze) often appears during beer aging, usually due to the interactions between proteins and polyphenols (e.g., catechin and epicatechin) within the beer [18,19,20]. The formation of protein–polyphenolic sediments could be one of the reasons behind the decreased content of phenolic compounds in beer observed after the aging stage. The total phenol content of the KVEIK-fermented beers produced in the present study ranged from 446.9 to 598.7 mg GAE/L. This is similar to the values determined by other authors in classic styles of top- and bottom-fermenting beers (lager, pilsner, wheat, and ale) [15]. Similar observations were made for the antioxidative activity determined in the FRAP test. The beers produced using KVEIK yeast showed ferric ion reducing capability of 3.54–4.14 mM TE/L, which is higher than the values reported for lager, pilsner, wheat, and ale style beers [15]. The KVEIK-type beers produced in the present study exhibited radical scavenging potential of 0.63–1.08 mM TE/L in the case of DPPH^•^ and 3.85–5.16 mM TE/L in the case of ABTS^•+^. Gouvinhas et al. (2021) and Zhao et al. (2010) analyzed the antioxidative potential of commercial beers using the above assays. The beers that they produced had higher DPPH^•^ and ABTS^•+^ scavenging activity than dark and light beers of ale and lager types [4]. In turn, compared to the results reported by Zhao et al. (2010), we produced beers with a similar antioxidative activity determined in the DPPH^•^ assay and higher activity measured in the ABTS^•+^ assay.

### 3.4. Content of Volatile Compounds

The GC–FID method used in the study allowed us to identify and quantify 21 volatile compounds in the beers produced with KVEIK yeast and in the control sample (Table 4). The largest group among the identified volatiles turned out to be alcohols (nine compounds). They were followed by esters (six compounds), aldehydes (four compounds), and two pyrazine group compounds. Among the analyzed beers, the VK2 and US-05 samples had the highest total content of volatile compounds (459.74 mg/L and 462.195 mg/L, respectively). The concentration of volatile compounds determined in the remaining beer samples was ca. 25% lower and reached 360.99 mg/L in LK beer, 361.16 mg/L in FM-53 beer, and 365.634 mg/L in HVK beer. The latter beer type had additionally the highest content of ethyl acetate (146.55 mg/L). A similar concentration of this compound was determined only in the control sample. Lower concentrations of this ester were determined in the FM53 and VK2 beers (101.77 and 107.58 mg/L, respectively), whereas its lowest concentration was determined in the LK sample, where it accounted for barely 60% of its content in the control sample (86.25 mg/L). The concentration of ethyl acetate, characterized by a fruity aroma, was above the detection level in all beer samples, and 3–5 times higher than in the bottom-fermenting beers analyzed by Hiralal, Pillay, and Olaniran (2013). A study conducted in 2017 by Preiss, Tyrawa, and van der Merwe demonstrated that some strains of KVEIK-type microorganisms could produce ethyl hexanoate, octanoate, and decanoate in light beers in amounts exceeding the human detection levels of 0.21 mg/L, 0.9 mg/L, and 0.20 mg/L, while, so far, no studies have been conducted on the concentration of these esters in dark beers. Only in the HVK beer was the content of ethyl hexanoate higher than the detection limit and reached 0.34 mg/L. The concentration of ethyl octanoate in KVEIK-fermented beers was below the detection limit (0.66–0.84 mg/L), while it was much higher in the control US-05 sample (4.60 mg/L). Ethyl decanoate was detected in each of the analyzed beers, and its content significantly exceeded the detection limit. The highest concentration of this ester (7.15–7.19 mg/L) was determined in the US-05 and HVK beer samples, and the lowest one in the FM-53 sample (1.99 mg/L). The so-called higher alcohols, which are often found in alcoholic beverages and can impart fruity aromas but also spirit or solvent aromas when present in excess concentrations to the aroma bouquet of fermented products, include such compounds as 2-methylbutanol, 3-methylbutanol, 1-propanol, or isobutanol [19]. The VK2 beer had the highest concentrations of 3-methylbutanol (154.64 mg/L), 2-methylbutanol (43.28 mg/L), and isobutanol (54.95 mg/L) among the analyzed beers, and all these compounds were determined in this sample at concentrations exceeding the detection limit. The HVK and LK beer samples were characterized by approximately 30–40% lower concentrations of 2-methylbutanol, 3-methylbutanol, and isobutanol than the control sample. In turn, the FM-53 sample had a similar concentration of isobutanol as in the control sample, despite significantly lower concentrations (the lowest of the analyzed beers) of 2-methylbutanol and 3-methylbutanol, and the highest concentration of 1-propanol (69.03 mg/L), being 74% higher than in the control beer. These compounds are produced in fermented beverages as a result of the metabolism of amino acids, such as isoleucine, valine, leucine, and alanine [20]. Differences noted in their concentrations despite using identical fermentation media (wort), which should have the same concentration of the above-mentioned amino acids, may indicate that KVEIK microorganisms are characterized by a different metabolism compared to typical brewer’s yeast, and that they show significant variety-dependent differences. Further differences in the metabolism of various substances from the brewing wort may be indicated by the trace amounts of some alcohols, such as 2-pentanol in the HVK, LK, and VK2 samples and 2-butanol in the LK sample, as these compounds were not identified in the control sample. In the HVK, LK, and VK2 beers, the concentration of toxic acetaldehyde (1.24–3.02 mg/L) was lower than in the US-05 beer (3.72 mg/L), which imparted a solvent aroma to alcoholic beverages. In turn, the FM-53 beer was characterized by an increased concentration of this compound (5.63 mg/L) [21]. A significant difference in the composition of volatile compounds in the tested samples lies also in the substantially higher concentration of phenethyl alcohol, characterized by a rose aroma and exhibiting bactericidal and fungicidal properties, which is widely used in the food and cosmetic industry. *Saccharomyces cerevisiae* yeast produces this compound de novo or by the bioconversion of phenylalanine, but the current research on biotechnological methods of phenethyl alcohol production has not revealed a viable medium or microorganism that would be suitable for its industrial-scale production [22]. In the LK and VK2 samples, its concentration reached 55.35 mg/L and 40.29 mg/L, respectively, which accounted for 182% and 133% of its content in the US-05 beer (30.30 mg/L), which suggests the potential of KVEIK microorganisms for its production.

### 3.5. Sensory Analysis

**Figure 1:**
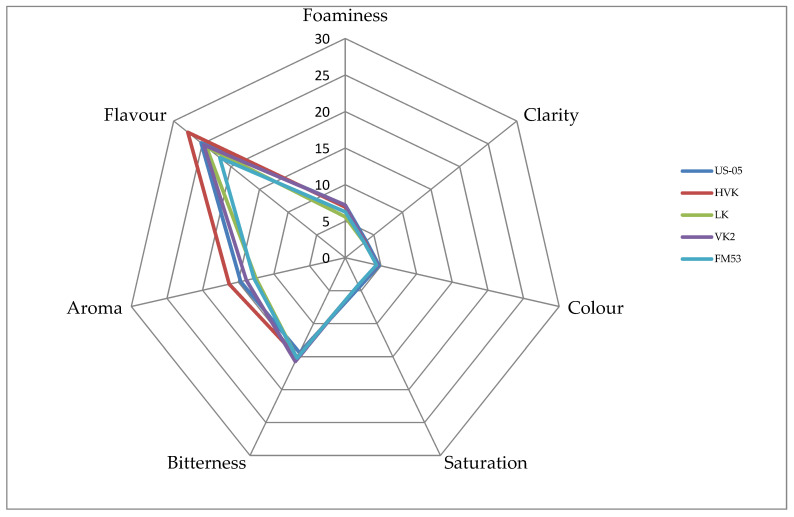
Results of the sensory analysis.

Beer analysis in terms of clarity, color, and saturation demonstrated that these quality attributes were rated by the panelists at the same level for the beers fermented using selected KVEIK yeast strains and the control beer fermented with a commercial yeast strain, US-05 (Figure 1). Foaminess was rated the highest in the KVEIK beer fermented with the VK2 strain and in the US-05 beer. A high but slightly lower score was also given by the panelists for foaminess to the LK beer. The bitterness of all beers tested was rated similarly; however, all KVEIK-fermented beers received one point more compared to the commercial US-05 beer. In the case of aroma, the highest score was given to the HVK beer, which received two points more than the US-05 beer and four points more than the beer fermented using Lida Kveik (LK) yeast. Taste, similar to the aroma, was rated the highest in the case of the HVK beer. It received two points more than the US-05 beer and three points more than the FM53 beer. Summing up, the beer that was best scored for all the assessed quality attributes turned out to be the one fermented with Horniwdak Var Kveik (HVK) yeast. It received three points more than the US-05 beer and nine points more than the FM53 beer.

In the descriptive beer analysis, the consumers indicated that the VK2 beer had a strange, metallic, smoked, and unpleasant taste. A higher number of comments concerned the FM53 beer, which was indicated as having a poorly perceptible aroma and shallow taste. Most of the consumers commented also on the beer fermented with the commercial US-05 yeast strain. It was indicated as having a very weak taste and aroma, particularly regarding fruity notes, which were strongly perceptible in the KVEIK beers.

The aroma of malt of all beers tested was evaluated by consumers as bread-like, coffee-like, roasted, and chocolate. In turn, the hop aroma was described as pine, resinous, and tea-like, whereas apricot, dried fruit, and apple were indicated among ester aromas. Among other aromas, the consumers also mentioned spicy, yeast-like, smoked, and woody. The bitterness intensity was assessed as moderate with a short finish, and its quality as mild. According to the consumers, the taste was dominated by coffee, chocolate, and roasted notes.

Recent research into beer preferences shows that consumers search for novel products with unusual flavors, often choosing craft beers that they believe have exceptional flavor and are of higher quality than commercial beers [23,24]. In order to meet customer expectations, beer producers have several options to develop different sensory profiles by, e.g., differentiating basic raw materials or additives, controlling the technological process, or selecting appropriate yeast strains [25,26,27]. The selection of the yeast strain is important in the context of the amount of aroma compounds formed. Therefore, unconventional yeast is increasingly used to produce beers with more complex sensory characteristics [28,29,30,31]. The distinguishing feature of KVEIK yeast is the production of above-threshold amounts of some esters, including ethyl caproate (tropical, pineapple), ethyl caprylate (apple, tropical, cognac), ethyl decanoate (apple), and isoamyl acetate (banana); hence, they can be successfully used in the production of fruity beers [1].

## 4. Conclusions

There were no significant differences in the consumption of glucose, maltose, and maltotriose between KVEIK yeast strains and the commercial yeast strain, which indicates that the yeast tested fulfilled an important technological criterion.

The mean concentration of ethyl alcohol and the mean fermentation degree determined in the KVEIK-fermented beers were similar to the values obtained in the control sample. This finding allows us to conclude that KVEIK yeast strains can successfully serve as alternative microorganisms to ferment malt wort.

The KVEIK yeast strains were capable of producing more glycerol than the commercial yeast strain, thereby improving the organoleptic traits of the finished product.

As the KVEIK yeast strains produced more glycerol and also fermented less dextrins than the US-05 strain, they can be successfully used to produce beers with a richer flavor (body).

The choice of the biological material can offer a viable method for increasing the concentration of phenolic compounds in beer and for boosting its antioxidative potential. After primary and secondary fermentation, all KVEIK-fermented beers had a higher or similar content of phenolic compounds to the control beer. After aging, a higher content of phenolics compared to the control sample was determined for LK, VK2, and FM53 beers. Ethanolic fermentation can lead to an increase in the total content of phenolic compounds, in the DPPH^•^ and ABTS^•+^ antioxidative activity, and in the ferric iron reducing capability of beers compared to the wort that they were produced with. The use of various types of brewer’s yeast affects the antioxidative properties of beers. The KVEIK-type yeasts, HVK and FM53, enabled the production of beers with higher antioxidative activity measured in the DPPH^•^ assay compared to the control sample. The highest antioxidative capacity measured in the ABTS^•+^ test was shown for the finished HVK beer, whereas the highest FRAP values were found for the finished FM53 and VK2 beers.

The beers fermented with the KVEIK-type yeast had different concentrations of many identified volatile compounds compared to the control sample. The LK, FM53, and HVK samples had lower concentrations of volatile compounds than the control beer, whereas VK2 beer had similar content of these compounds. The major volatile compounds identified in the study included ethyl acetate and hexanate in the HVK sample; phenethyl alcohol in the LK beer sample; 2-methylbutanol and 3-methyle butanol in the VK2 sample; 1-propanol in the FM53 beer sample; and, finally, ethyl octanoate and decanoate in the control US-05 sample.

The sensory panel had greater preferences for beers fermented with KVEIK yeast due to the more intense fruity aroma. The degree of bitterness was also rated more highly in relation to beers fermented with a commercial yeast strain. The best-rated beer in terms of flavor and aroma was the one fermented with HVK.

## Figures and Tables

**Table 1:** Basic physicochemical parameters.

Sample	Stage of brewing	Alcohol	Alcohol	Real Extract	Apparent Extract	RDF	ADF	Energy Value	pH
		% *v*/*v*	% *w*/*w*	% *w*/*w*	% *w*/*w*	%	%	kcal/100 mL	
	Wort	nd ^1^	nd	11.08 ± 0.03 a	10.75 ± 0 a	nd	nd	44.12 ± 0 cdef	5.85 ± 0
HVK	After primary fermentation	3.56 ± 0.2 b ^2^	2.79 ± 0.19 b	7.16 ± 0 ab	5.39 ± 0 ab	49.69 ± 0.29 a	61.63 ± 0.36 a	43.35 ± 1.39 b	5.07 ± 0.113 a
LK		3.60 ± 0.06 b	2.76 ± 0.06 b	7.24 ± 0.06 ab	5.34 ± 0.03 ab	50.07 ± 0.06 a	62.09 ± 0.07 a	43.53 ± 0.64 b	5 ± 0.311 a
VK2		3.87 ± 0.52 ab	2.84 ± 0.23 b	7.30 ± 0.04 a	5.48 ± 0.01 a	49.53 ± 0.21 a	61.43 ± 0.25 a	43.61 ± 1.13 b	5.05 ± 0.198 a
FM53		3.78 ± 0.28 ab	2.97 ± 0.25 b	7.03 ± 0.16 bc	5.24 ± 0.15 bc	49.91 ± 0.15 a	61.89 ± 0.18 a	45.21 ± 1.7 b	5.17 ± 0.198 a
US-05		4.51 ± 0.1 a	3.47 ± 0.08 a	6.91 ± 0 c	5.14 ± 0.06 c	50.06 ± 0.46 a	62.73 ± 1.46 a	48.79 ± 0.62 a	4.98 ± 0.325 a
HVK	After secondary fermentation	5.06 ± 0.09 a	3.91 ± 0.07 a	7.13 ± 0.08 a	5.50 ± 0.01 ab	51.21 ± 0.18 b	63.5 ± 0.22 b	39.08 ± 1.53 b	4.54 ± 0.021 b
LK		5.25 ± 0.01 a	4.06 ± 0.00 a	7.39 ± 0.06 a	5.64 ± 0.08 ab	51.14 ± 0.2 b	63.41 ± 0.24 b	40.68 ± 2.72 b	4.64 ± 0.071 ab
VK2		5.26 ± 0.21 a	4.07 ± 0.15 a	7.48 ± 0.25 a	5.68 ± 0.16 a	50.88 ± 0.05 b	63.08 ± 0.06 b	42.36 ± 1.55 ab	4.65 ± 0.057 ab
FM53		5.25 ± 0.08 a	4.07 ± 0.06 a	7.44 ± 0.03 a	5.71 ± 0.06 a	50.97 ± 0.5 b	63.2 ± 0.62 b	42.14 ± 0.2 ab	4.66 ± 0.064 ab
US-05		5.25 ± 0.08 a	4.07 ± 0.06 a	7.18 ± 0.16 a	5.41 ± 0.08 b	51.94 ± 0.21 a	64.39 ± 0.25 a	46.74 ± 2.67 a	4.77 ± 0.049 a
HVK	After aging	5.11 ± 0.01 b	3.97 ± 0.01 b	6.66 ± 0.28 a	4.91 ± 0.33 a	52.75 ± 1.07 c	64.79 ± 2.2 c	44.3 ± 0.52 b	4.53 ± 0.028 b
LK		5.58 ± 0.06 a	4.20 ± 0.01 a	5.05 ± 0.59 c	3.05 ± 0.29 c	62.51 ± 2.62 a	77.41 ± 3.24 a	41.8 ± 1.96 b	4.74 ± 0.035 ab
VK2		5.31 ± 0.15 b	4.11 ± 0.09 ab	6.57 ± 0.06 ab	4.78 ± 0.04 a	53.2 ± 0.04 c	65.94 ± 0.04 bc	45.97 ± 1.55 ab	4.77 ± 0.170 ab
FM53		5.30 ± 0.14 b	4.10 ± 0.11 ab	5.61 ± 0.56 bc	3.76 ± 0 b	58.54 ± 2.34 ab	72.02 ± 3.47 ab	42.45 ± 1.46 b	4.65 ± 0.071 ab
US-05		5.27 ± 0.08 b	4.07 ± 0.06 ab	6.30 ± 0.14 ab	4.43 ± 0.18 a	55.33 ± 1.15 bc	68.57 ± 1.42 bc	49.78 ± 2.03 a	4.83 ± 0.071 a

^1^ nd, not detected. ^2^ Values are expressed as the mean (*n* = 3) ± standard deviation. Mean values with different letters (a, b, c, etc.) within the same row are statistically different (*p*-value < 0.05).

**Table 2:** Concentrations of carbohydrates and glycerol in the analyzed wort and beers.

Beer	Stage of Brewing	Dextrins	Maltotriose	Maltose	Glucose	Lactic Acid	Glycerol	Acetic Acid
		g/L	g/L	g/L	g/L	g/L	g/L	g/L
	Wort	42.11 ± 0.29 a ^1^	15.32 ± 0.13 a	45.25 ± 0.367 a	8.06 ± 0.11 a	nd ^2^	nd	nd
HVK	After primary fermentation	39.74 ± 1.04 a	nd	nd	nd	nd	1.27 ± 0.05 b	nd
LK		40.61 ± 0.86 a	nd	nd	nd	nd	1.32 ± 0.04 b	nd
VK2		41.25 ± 0.35 a	2.74 ± 0.36	nd	nd	nd	1.45 ± 0.01 a	nd
FM53		40.82 ± 1.49 a	nd	nd	nd	nd	1.43 ± 0.03 a	nd
US-05		41.21 ± 0.61 a	nd	nd	nd	nd	1.31 ± 0.01 b	nd
HVK	After secondary fermentation	27.94 ± 0.78 d	nd	nd	nd	nd	0.96 ± 0.09 d	nd
LK		39.05 ± 1.20 b	nd	nd	nd	nd	1.25 ± 0.03 b	nd
VK2		41.25 ± 0.35 a	nd	nd	nd	nd	1.44 ± 0.00 a	nd
FM53		28.49 ± 0.93 d	nd	nd	nd	nd	1.45 ± 0.03 a	nd
US-05		35.02 ± 0.10 c	nd	nd	nd	nd	1.11 ± 0.03 c	nd
HVK	After aging	37.18 ± 0.71 b	nd	nd	nd	nd	1.38 ± 0.02 b	nd
LK		40.62 ± 1.06 a	nd	nd	nd	nd	1.61 ± 0.01 a	nd
VK2		40.93 ± 0.28 a	nd	nd	nd	nd	1.66 ± 0.02 a	nd
FM53		37.43 ± 0.78 b	nd	nd	nd	nd	1.39 ± 0.00 b	nd
US-05		35.65 ± 0.90 b	nd	nd	nd	nd	1.12 ± 0.03 c	nd

^1^ Values are expressed as the mean (*n* = 3) ± standard deviation. Mean values with different letters (a, b, c, etc.) within the same row are statistically different (*p*-value < 0.05). ^2^ nd, not detected.

**Table 3:** Total polyphenol content and antioxidant activity of wort and beers.

Sample	TPC	DPPH·	ABTS·+	FRAP
	mg GAE/L	mM TE/L		
Wort				
W	447.9 ± 7.0 f ^1^	0.39 ± 0.072 g	3.26 ± 0.38 g	1.43 ± 0.09 h
Beer after primary fermentation				
US-05	493.2 ± 17.9 e	0.86 ± 0.088 cd	5.32 ± 0.29 ab	2.88 ± 0.2 f
HVK	586.7 ± 19.7 c	0.62 ± 0.048 ef	5.64 ± 0.14 a	2.92 ± 0.07 ef
LK	495.3 ± 7.8 e	0.97 ± 0.058 bc	5.75 ± 0.19 a	2.62 ± 0.13 g
VK2	710.7 ± 20.7 a	1.29 ± 0.024 a	5.3 ± 0.32 abc	3.4 ± 0.13 cd
FM53	554.2 ± 6.40 d	0.93 ± 0.02 c	5.3 ± 0.1 abc	2.57 ± 0.13 g
Beer after secondary fermentation				
US-05	594.5 ± 11.9 c	0.63 ± 0.093 ef	3.38 ± 0.32 g	3.23 ± 0.21 d
HVK	625.1 ± 24.2 b	0.61 ± 0.091 f	5.01 ± 0.55 bcd	3.2 ± 0.27 d
LK	631.4 ± 11.5 b	0.59 ± 0.057 f	4.59 ± 0.43de	3.23 ± 0.2 d
VK2	648.2 ± 9.3 b	0.42 ± 0.056 g	4.39 ± 0.36 ef	3.55 ± 0.19 c
FM53	636.3 ± 2.5 b	0.56 ± 0.044 f	3.69 ± 0.23 g	3.15 ± 0.08 de
Beer after aging				
US-05	466.1 ± 17.5 f	0.62 ± 0.111 ef	4.68 ± 0.19 cde	3.57 ± 0.09 c
HVK	446.9 ± 7.8 f	1.08 ± 0.097 b	5.16 ± 0.39 abcd	3.54 ± 0.16 c
LK	561.3 ± 16.2 d	0.63 ± 0.06 ef	3.85 ± 0.28 fg	3.87 ± 0.1 b
VK2	560.1 ± 11.6 d	0.74 ± 0.051 de	4.72 ± 0.55 bcde	3.98 ± 0.07 ab
FM53	598.7 ± 14.8 c	0.79 ± 0.035 d	4.35 ± 0.2 ef	4.14 ± 0.05 a

^1^ Values are expressed as the mean (*n* = 3) ± standard deviation. Mean values with different letters (a, b, c, etc.) within the same row are statistically different (*p*-value < 0.05).

**Table 4:** Concentration of volatile compounds in beers produced with the use of KVEIK yeast.

Nr	Compound ^1,2^	Chemical Group	US-05	HVK	LK	VK2	FM-53
			(mg/L)				
1	2-butanol	Alcohols	nd	nd	0.37 ± 0.18 a	nd	nd
2	1-propanol	Alcohols	39.64 ± 1.14 c	30.89 ± 0.99 d	27.81 ± 0.73 e	41.57 ± 1.34 b	69.03 ± 1.11 a
3	Isobutanol	Alcohols	50.73 ± 1.42 c	31.90 ± 0.74 d	33.09 ± 0.93 d	54.95 ± 1.22 ab	52.84 ± 0.72 bc
4	2-pentanol	Alcohols	trace	0.21 ± 0.07 a	0.08 ± 0.02 b	0.01 ± 0.01 b	trace
5	1-butanol	Alcohols	0.88 ± 0.04 a	0.58 ± 0.03 c	0.70 ± 0.01 b	0.69 ± 0.03 b	0.20 ± 0.01 d
6	2-methylbutanol	Alcohols	36.93 ± 1.39 b	25.77 ± 0.55 cd	28.71 ± 0.66 c	43.28 ± 1.03 a	24.69 ± 0.38 d
7	3-methylbutanol	Alcohols	140.30 ± 3.75 b	101.76 ± 2.84 d	108.68 ± 3.51 c	154.64 ± 3.54 a	74.52 ± 1.05 e
8	1-hexanol	Alcohols	0.04 ± 0.02 ab	0.01 ± 0.01 c	0.02 ± 0.01 abc	0.04 ± 0.01 ab	0.05 ± 0.02 ab
9	Phenylethyl alcohol	Alcohols	30.30 ± 0.55 bc	28.90 ± 5.05 bc	55.35 ± 6.50 a	40.29 ± 2.73 b	23.56 ± 1.16 c
10	Acetaldehyde	Aldehydes	3.72 ± 0.07 b	1.24 ± 0.62 d	2.75 ± 0.09 c	3.02 ± 0.47 c	5.63 ± 0.09 a
11	Propanal	Aldehydes	0.33 ± 0.01 c	0.73 ± 0.02 a	0.52 ± 0.14 b	0.33 ± 0.01 c	0.24 ± 0.12 d
12	Hexanal	Aldehydes	0.065 ± 0.004 b	0.074 ± 0.002 a	nd	nd	nd
13	Furfural	Aldehydes	4.55 ± 2.54 c	13.97 ± 1.88 a	11.58 ± 1.26 ab	7.79 ± 0.44 bc	4.78 ± 0.14 c
14	Ethyl acetate	Esters	142.01 ± 2.56 b	146.55 ± 1.73 a	86.25 ± 2.69 e	107.58 ± 0.80 c	101.77 ± 0.41 d
15	Ethyl isobutyrate	Esters	0.03 ± 0.02 b	0.07 ± 0.01 a	nd	nd	nd
16	Isopentyl acetate	Esters	0.51 ± 0.01 d	0.52 ± 0.01 d	0.82 ± 0.01 a	0.65 ± 0.01 b	0.60 ± 0.02 c
17	Ethyl hexanoate	Esters	0.17 ± 0.03 b	0.34 ± 0.01 a	0.20 ± 0.03 b	0.17 ± 0.02 b	0.19 ± 0.02 b
18	Ethyl octanoate	Esters	4.60 ± 3.81 a	0.84 ± 0.01 b	0.68 ± 0.01 b	0.70 ± 0.01 b	0.66 ± 0.01 b
19	Ethyl decanoate	Esters	7.15 ± 0.11 a	7.19 ± 0.21 a	3.10 ± 0.19 b	3.94 ± 0.14 b	1.99 ± 0.12c
20	2,5-dimethylpyrazine	Pyrazines	0.24 ± 0.09 a	0.10 ± 0.06 b	0.28 ± 0.01 a	0.06 ± 0.02 b	0.07 ± 0.04 b
21	2,3-dimethylpyrazine	Pyrazines	nd	nd	trace	0.03 ± 0.02b	0.34 ± 0.05 a
	Total volatiles		462.195	365.634	360.99	459.74	361.16

^1^ Values are expressed as the mean (*n* = 2) ± standard deviation. Mean values with different letters (a, b, c, d, e) within the same row are statistically different (*p*-value < 0.05). ^2^ nd, not detected.

## Data Availability

The data presented in this study are available on request from the corresponding author.
